# Magendie’s Physiology

**Published:** 1844-03

**Authors:** 


### MAGENDIE'S PHYSIOLOGY.

We owe a copy of this work, a new edition of which he has given to the public, through the Harpers, to the politeness of the editor, Professor Revere. Magendie is one of the most distinguished of living physiologists, and his work has this excellence, that it is not a mere summary of the doctrines and doings of other men in this department of science. He is an original investigator, and in regard to the performance of experiments on living animals has surpassed all other physiologists. In fact, he has been thought to have carried these experiments farther than humanity justified, or the interests of science demanded. But it cannot be denied, that his untiring industry in the prosecution of such researches has enlarged the science of physiology, and contributed to the revolution which it has undergone, on many points, since the first edition of his Summary was published. As remarked by the editor, Magendie's work has this other adimirable feature, that it pursues a "severe system of induction, excluding those imaginative and speculative views, which rather belong to metaphysics than physiology." The style, as that of such works ought to be, is concise and perspicuous, its descriptions are marked by clearness, and its well-arranged matter is brought within the compass of a single volume of moderate dimensions.

The book is for sale at the store of J. Maxwell, jr., and of Morton & Griswold, book-sellers, in this city. Y.

